# Fixation of olecranon osteotomy only with 6′5 mm partially trheaded cancellous screw is a safe an effective method used in surgical management of distal humerus fractures

**DOI:** 10.1186/s40634-020-00317-8

**Published:** 2021-01-12

**Authors:** Pablo Cañete San Pastor, Javier Lopez Valenciano, Ivan Copete, Inma Prosper Ramos

**Affiliations:** Orthopaedic Surgery Department, Hospital de Manises, Valencia, Spain

**Keywords:** Supraintercondylar humeral fractures, Olecranon osteotomy, Distal humeral fractures, Trauma

## Abstract

**Purpose:**

The objective of this study is to demonstrate the safety and efficacy of the osteosynthesis with a 6.5 mm screw and washer of a Chevron shape olecranon osteotomy performed for the surgical approach of supraintercondylar fractures of the distal humerus, achieving union and complication rates better or similar to other published case series.

**Methods:**

From 2009 to 2019, 26 patients underwent fixation of an olecranon osteotomy for the treatment of a supraintercondylar fracture of the distal humerus with partially threaded cancellous cannulated screws of 6.5 mm diameter with a washer. The patients were followed for at least 1 year, taking radiographs the day after the surgery, at 3, 6 and 12 months. Complications have been collected: infection, loss of reduction, non-union, delay of union, discomfort of the osteosynthesis hardware.

The diameter of the ulna medullary canal diaphysis was also measured in all patients.

**Results:**

Consolidation of the osteotomy was 100% at 12 months. The average time of radiological consolidation was 112 ± 12 days. The average size of the ulna medullary canal diaphysis was 6′06 ± 0′16 mm on anteroposterior radiographs and 5′65 ± 0′14 mm on lateral radiographs. The mean screw length was 102′31 mm ± 3′89. We found 1 acute infection, 2 osteotomies delays of union (one of these cases was the acute infection case), one early osteosynthesis failure and 1 wound dehiscence.

**Conclusions:**

Olecranon ostetomy fixation with a 6′5 mm cancelous partial threaded screw and washer is safe and effective with a high consolidation rate and excellent results and with complication rates similar to or lower than other fixation methods published. Long enough screws must be used to get a good cortical grip with enough stability.

**Level of evidence:**

Level IV, Case series, retrospective review.

## Introduction

Joint fractures of the distal humerus account for 2% of all fractures [6]. The treatment of displaced fractures is usually surgical, being open reduction and internal fixation the most used method; there is also the option of orthopaedic treatment or an elbow prosthesis in cases of fracture with great joint conminution in elderly patients [8, 11, 13].

A good surgical approach is important in order to achieve an anatomical reduction of the joint and a stable fixation to initiate early mobility and have good outcomes. Although different surgical approaches have been described: medial and lateral paratricipital approach, triceps sparing, triceps split, etc., the olecranon osteotomy is the most frequent and the one that provides the best vision and approach to fracture [4, 14, 16, 21].

The Chevron V osteotomy with distal vertex is the most widely used; increases the stability of the osteotomy and decreases the rate of complications. There are different ways to fix the osteotomy: tension band, plate fixation and hybrid constructs with screws are the most commonly used [7, 9, 10, 16, 17, 20]. The main complications of osteotomy are non-union, poor consolidation with loss of joint reduction, and hardware discomfort that requires removal [7, 9, 10, 13, 17, 21].

Authors such as Ring in 2004 [17] obtained good results with the osteotomy fixation with tension band with Kirschner wires penetrating the anterior cortex of the ulna. In 2015 Wagener [19] used intramedullary cancellous screw and suture tension band with very good results in 19 patients. Iorio uses a novel device (the olecranon sled), obtains excellent rates of union [10].

Woods in 2015 [21] demonstrated that the fixation of the ostetomy with screw and washer was an effective and safe method: They retrospectively reviewed one hundred sixty patients with distal humerus fracture treated operatively with an olecranon osteotomy for the exposure; 39 patients underwent screw fixation alone, 47 had a tension band fixation, 16 had plate fixation, and 58 had tension band and screw fixation. They conclude that screw fixation demonstrated equal or better rates of union, maintenance of reduction, absence of infection, and implant removal compared with alternative fixation techniques.

In our study, we have reviewed the patients operated for distal humerus fracture with whom we have performed a chevron osteotomy of the olecranon for exposure and we have fixed it with a 6.5 mm diameter partially threaded cancellous screw with a washer, long enough to grip the distal cortex of the ulna. The objective of our technique is to achieve interfragmentary compression in the fixation of the olecranon osteotomy, getting a higher rate of fracture consolidation and a great initial stability that allows early mobility and loading of the elbow.

The specific anatomy of the ulna, with a narrow canal and varus bow that often exists, allows an endomedullary screw of sufficient diameter and length to grip cortical bone. We have measured the canal of the diaphysis of the ulna to verify that with a 6.5 mm screw of sufficient length we can obtain a distal cortical fixation, thus achieving our goal of interfragmentary compression and stable fixation.

The objective of our work is to demonstrate that with a screw of sufficient diameter and length with a washer we get enough compression and stability to achieve a high rate of consolidation and early mobility and loading in the olecranon osteotomy performed in the approach to fractures of the distal humerus.

Our hypothesis is that with this technique we obtain a safe and stable fixation with rates of olecranon union and complications equal or better than with other published techniques.

## Material and methods

From 2009 to 2019, we have treated 54 supracondylar fractures of the distal humerus. An osteotomy of the olecranon was performed for the approach in 43 cases, fixed with plates, tension band, screws and tension band, or screws only. A longitudinal, retrospective study was conducted on 26 patients with a supraintercondylar fracture of the humerus treated with an approach using a V chevron olecranon osteotomy fixed only with partially threaded cancellous cannulated screws of 6.5 mm diameter with a washer (Zimmer-Biomet, Warsaw, Indiana).

The following data have been collected: age, sex, fracture side, year of the surgery, narrow marrow ulnar diaphysis diameter, follow up time, screw length, type of humerus osteosynthesis, time of osteotomy consolidation, complications, screw removal, reoperation (Table 1).

**Table 1:** Data of patients

**Patient number**	**Surgery Year**	**Age**	**Side**	**Screw size**	**Adjuvant**	**AP Humerus**	**L. Humerus**	**Early**	**Complications**	**Radiographic**	**Material exeresis**	**Time of exeresis**
				**(mm)**	**osteosynthesis**	**width (cm)**	**width (cm)**	**complications**		**consolidation (months)**		**(months)**
**1**	2009	26	Right	85	Washer	6.25	5.95	No	Discomfort plate	3	Yes	6
**2**	2010	71	Right	65	Washer	5.50	4.40	Consolidation delay	Infection (surgical cleaning)	6	No	
**3**	2010	73	Right	105	Washer	8.50	8.00	No	Discomfort plate	3	Yes	12
**4**	2011	76	Right	90	Washer	5.60	5.20	No	No	3	No	
**5**	2011	25	Right	115	Washer	4.90	5.00	No	No	3	Yes	12
**6**	2011	46	Left	130	Washer	5.70	5.18	No	No	3	No	
**7**	2012	73	Right	120	Washer	5.70	4.90	Osteosynthesis failure	Osteosynthesis failure (revision surgery)	3	No	
**8**	2012	16	Left	90	Washer	5.08	6.00	No	Discomfort plate	3	Yes	13
**9**	2012	78	Left	110	Washer	6.10	4.70	No	No	4	No	
**10**	2013	18	Left	100	Washer	5.90	5.63	No	No	3	No	
**11**	2013	99	Right	100	Washer	5.78	5.40	No	No	3	No	
**12**	2014	63	Right	125	Washer	5.86	6.10	No	No	3	No	
**13**	2014	66	Right	130	Washer	5.75	5.80	No	No	3	No	
**14**	2014	30	Left	110	Washer	5.96	5.74	No	No	3	No	
**15**	2015	83	Right	90	Washer	6.84	5.18	No	No	3	No	
**16**	2017	32	Left	100	Washer	5.50	5.15	No	Discomfort plate	3	Yes	12
**17**	2017	16	Left	80	Washer	6.50	6.80	No	Discomfort plate	3	Yes	10
**18**	2018	19	Right	105	Washer	5.30	5.20	No	Discomfort plate	3	Yes	10
**19**	2018	59	Left	120	Washer	6.05	6.00	No	No	3	No	
**20**	2018	78	Right	100	Washer	7.00	5.80	No	No	3	No	
**21**	2018	62	Right	85	Washer	7.50	6.50	No	No	3	No	
**22**	2019	60	Left	95	Washer	6.26	5.95	Consolidation delay	Consolidation delay	12	No	
**23**	2019	23	Right	115	Washer	5.20	5.00	No	Discomfort plate	3	Yes	12
**24**	2019	83	Left	115	Washer	7.20	5.50	Wound dehiscence	Wound dehiscence	3	Yes	2´5 (screw)
**25**	2019	62	Right	90	Washer	6.42	6.20	No	No	3	No	
**26**	2019	89	Left	90	Washer	5.40	5.60	No	No	3	No	

Skeletally inmature patients, patients without olecranon osteotomy, patients with another method of osteotomy fixation, pathological fractures and lost to follow up patients have been excluded.

The mean age of the patients is 54.84 ± 5.13 years [Range 16–99].

The 26 patients were operated by the same shoulder and elbow surgeon. All the patients have been followed for at least 1 year, taking radiographs the day after the surgery, at 3, 6 and 12 months. Complications have been collected: infection, loss of reduction, non-union, delay of union, discomfort of the osteosynthesis hardware. The osteotomy non-union was defined as the presence of radiolucency on x-ray 9 months after surgery [20]; and delay of union, radiolucency at 3 months. More than 2 mm of step-off or gap after fixation of the osteotomy was considered like loss of reduction [5, 10, 17, 21].

**Fig. 1:**
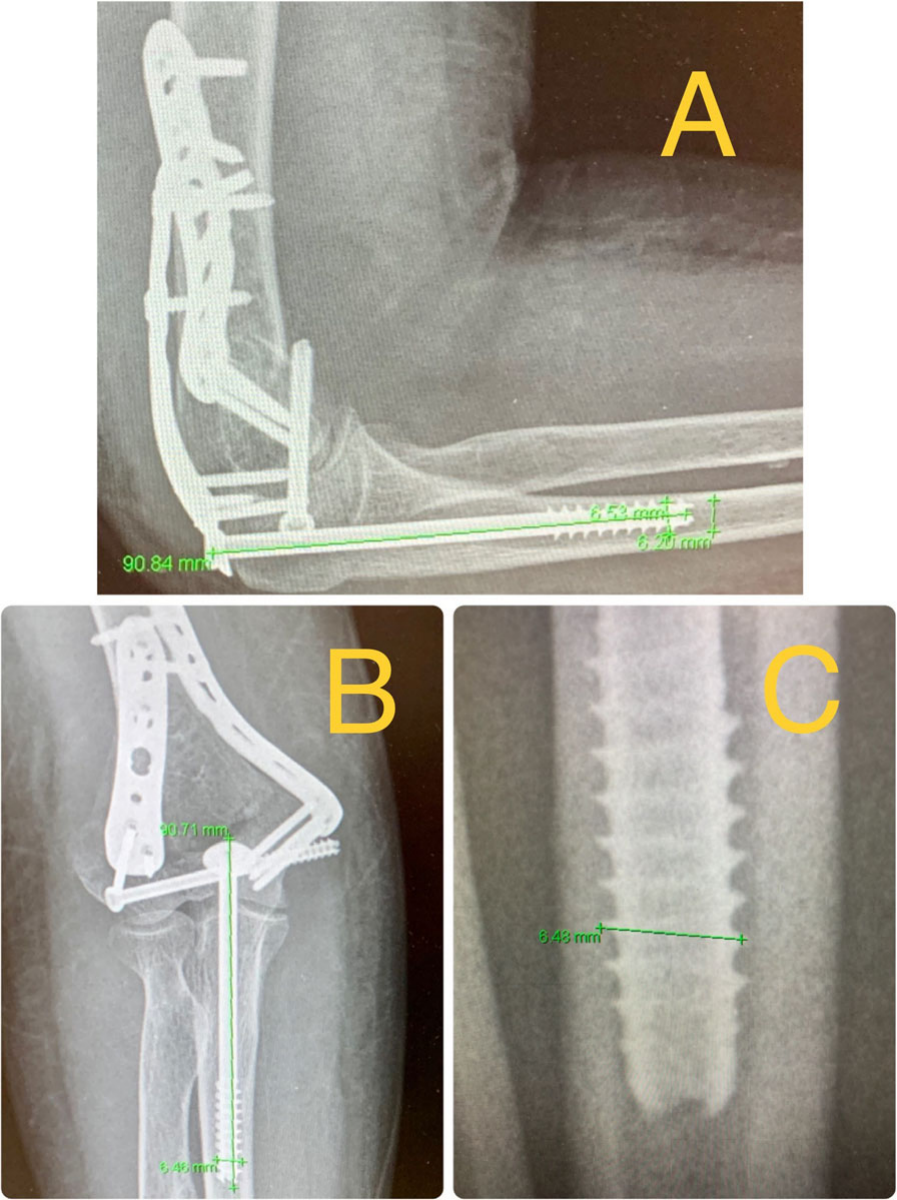
**a**, **b** 63 year old man who presented a conminuted supraintercondylear fracture of the humerus. Osteosynthesis of the fracture with two plates and a 6.5 mm thick and 90 mm long screw with washer for the olecranon osteotomy. The diameter of the ulna medullary canal diaphysis at about 9 cm from the tip of the olecranon were mesured using the measuring system of the program by Centricity Enterprise Web 3.0 DICOM conformance Memo (GE Medical Systems). The results in this case were 6′42 mm in the AP view and 6′2 mm in the lateral view. The diameter and the length of the 6′5 mm screw (90 mm in this case) were measured using the measurement system, to check the results and take it as a valid reference for the measurement of the diameter of the ulna medullary canal diaphysis. **c** Screw diameter measurement of 6′5 mm. The zoom tool of the system can be applied without altering the measurement, which make it easier

The diameter of the ulna medullary canal diaphysis has been measured, at about 90 mm from the tip of the olecranon (the area where the anteromedial curve starts) using the measuring system of the program by Centricity Enterprise Web 3.0 DICOM conformance Memo (GE Medical Systems). All the measurements were made by the same observer; Previously checking that the diameter of the screw measured 6.5 mm, and that the length of the screw, agreed with the length of the implanted screw (recorded in the surgical history of the patient), in order to take it as a valid reference for the measurement of the diameter of the ulna medullary canal diaphysis (Fig. 1).

The ulnar diameter measurements were made in the pre-operative X-ray of the fracture. The measurement is more reliable in the lateral X-ray, since, on many occasions, the anteroposterior one is not performed with the elbow fully extended, which distorts the measurement.

### Surgical technique

The vertex of the osteotomy is marked on the dorsal side of the ulna and the osteotomy is designed in a V shape with a distal vertex. Once the osteotomy is marked with a cautery device, and before carrying it out, the osteosynthesis screw is implanted. For this, with radioscopic control, a 2.8 mm intramedullary guide wire is inserted from the tip of the olecranon, ensuring that it is centred in the anteroposterior and lateral plane. A cannulated 4.8 mm drill bit is then inserted until it feels it has reached the cortex, then it is advanced at least 1 cm more.

A measurement is made of what will be the length of the cancellous partial threaded 6.5 mm screw (Zimmer-Biomet, Warsaw, Indiana). The screw is then inserted, checking that it achieves a good grip in the cortex of the ulnar diaphysis. The screw is extracted and is saved in order to insert it at the end of the surgery. The osteotomy is performed. The osteotomy is started with an oscillating saw and completed with a chisel, which achieves a certain irregularity in the osteotomy and increases stability. This also takes into account, that in the dorsal cortex there will be a small gap of 1 mm due to the bone that is lost on performing the osteotomy with the saw [10, 17, 21].

After completing the osteotomy, the olecranon and the triceps are brought up to the proximal, having an excellent view of the distal humerus.

Once the synthesis of the humerus is finished, the olecranon is moved to its anatomical position. The 2.8 mm guide wire is passed through the hole of both bone fragments of the olecranon and a bone clamp is put in place in order to maintain the osteotomy reduction.

The anatomical reduction is checked by visualising the articular part, the naked area, since it has already been mentioned the dorsal part remains slightly separated due to the loss of bone on performing the osteotomy with the saw. The partially threaded cancellous cannulated 6.5 mm screw is inserted initially with a motor machine and the last turns performed manually to check for a good grip, the compression, and the stability.

At the end of the surgery, a compression bandage is placed, which is removed after 48 h, and the patient is encouraged to begin active, self-assisted mobility of the elbow. Rehabilitation begins 2 weeks after removal of the stitches. Patients are allowed for early load and mobility of the elbow; also early weight bearing if needed (this injury can present in a politrauma patient that need to use crutches).

Muscle strengthening and heavy weight bearing exercises start of the second month.

### Statistical analysis

Statistical analysis was performed with SPSS statistics v.22 © (IBM). Initially, the sample normality was assessed with a Kolgomorov-Smirnoff study and Lilliefors correction, proving that although age followed a normal distribution, the rest of the variables did not have a binomial distribution. Therefore, for the analysis of the quantitative variables we used the ANOVA test. In non parametric variables we used the U Mann–Whitney test. For the analysis of qualitative variables we use the chi-square test. A statistical difference with a p value of *p* < 0.05 was considered significant.

## Results

Average age of the 26 patients was 54′84 ± 5′13 years (Range 16–89). 57′7% were younger than 64 years and 57′7% of the cases presented in right elbows (Table 1). It was found that age did not influence the osteotomy healing time and the presence of major complications. However, a younger age was significantly related to presenting minor complications such as discomfort with the osteosynthesis hardware (*p* = 0.030) and with the rate of hardware removal (*p* = 0.001).

Osteosynthesis: The mean screw length was 102′31 mm ± 3′89 (65–130 mm).

Regarding osteosynthesis in the humerus, in 21 cases 2 plates (medial and lateral) were used, in 4 cases only a lateral plate and in 1 case just a medial plate. In 46.4% of cases, additional fixation hardware (3′5 mm interfragmentary screws or countersinkable compression were used to increase stability. The type of osteosynthesis used in the humerus was not related to the consolidation of the olecranon osteotomy.

The average size of the ulna medullary canal diaphysis 9 cm from the tip of the olecranon was 6.06 ± 0.16 mm on anteroposterior radiographs and 5.65 ± 0.14 mm on lateral radiographs. Surprisingly, there was no correlation between screw length for olecranon osteosynthesis and ulna medullary canal diaphysis in either plane of the x-rays.

The average time of radiological consolidation was 112 ± 12 days.

Consolidation was 100% at 12 months. We can verify that the size of the screw did not influence the consolidation time (*p* = 0.288).

### Complications

84,6% of the patients didn’t present complications during the first 3 months (early complications). Among the complications, we found 1 acute infection; 2 osteotomies delays of union (one of these cases was the acute infection case); one early osteosynthesis failure (probably due to technical mistake) that required reoperation at 2 days changing the osteosynthesis to a tension band cerclage with union of the osteotomy at 2 months and good functional result (Fig. 3); and 1 wound dehiscence that required outpatient nursing cures every 48 h and the removal of the 6.5 mm screw at 10 weeks after the surgery, when bone callus was observed in most part of the ulna, considering the bone union stable (Fig. 4); the wound healed well after the screw removal (Table 1).

During the follow-up, we divided the complications into major and minor.

Only 2 patients presented major complications: an acute infection that required surgical cleaning without hardware removal and that consolidated at 6 months; and the acute osteosynthesis failure previously mentioned.

Minor complications were more frequent, 26.9% (7/26) of the patients presented discomfort in the hardware osteosynthesis (although the discomfort was mainly in the osteosynthesis plates of the distal humerus); 1 asymptomatic delay of union and one wound dehiscence previously discussed. Patients who presented early complications (in the first 3 months) were very likely to have both major and minor complications in the long term, *p* < 0.005.

Only 8 of the 26 patients requested hardware removal (mainly the osteosynthesis plates of the humerus), one of them without presenting any discomfort. The mean time for removing the hardware was 9.94 ± 1.16 months. This fact was statistically significant with the presence of minor complications, especially discomfort in the osteosynthesis material (*p* < 0.005).

Even so, 65.4% of the patients did not present complications. The presence of complications did not predict the need to subsequently hardware removal.

## Discussion

In our work, we have shown that with a screw of sufficient diameter (6.5 mm) and length (102 mm on average) and a washer, we achieve stable fixation in the olecranon osteotomy performed for the approach of supracondylar humerus fractures. This allows us an early mobility and loading in patients; and obtain a consolidation of 100% of the cases, with a low rate of complications.

When we are faced with any supracondylar elbow joint fracture, the objective will be an anatomical reduction and a stable fixation that allows early mobilization in order to obtain the best possible clinical and functional outcome [8, 11, 13]. In order to achieve this objective, an approach that provides good access to the joint is needed. In 2019 Ramsey put a plate in the olecranon before the osteotomy and then he used a Gigli saw, passing it anterior to the olecranon and using it to create an osteotomy through the bare area of the sigmoid notch. He presented good results in 5 cases [16].

According to the literature and our experience, the best way of doing the olecranon osteotomy is with a V-shaped Chevron osteotomy, although this has certain risks [4, 14, 15, 19, 20]. The technique that we present manages to reduce these risks by achieving a stable fixation with a minimum of osteosynthesis hardware that does not protrude and is a quick, safe, straightforward, and reproducible technique, at the end of a generally long surgery (150 min on average according to Coles [5] or up to 5 h according to Russell [18]), with a tired surgeon and with a higher risk of making mistakes.

**Fig. 2:**
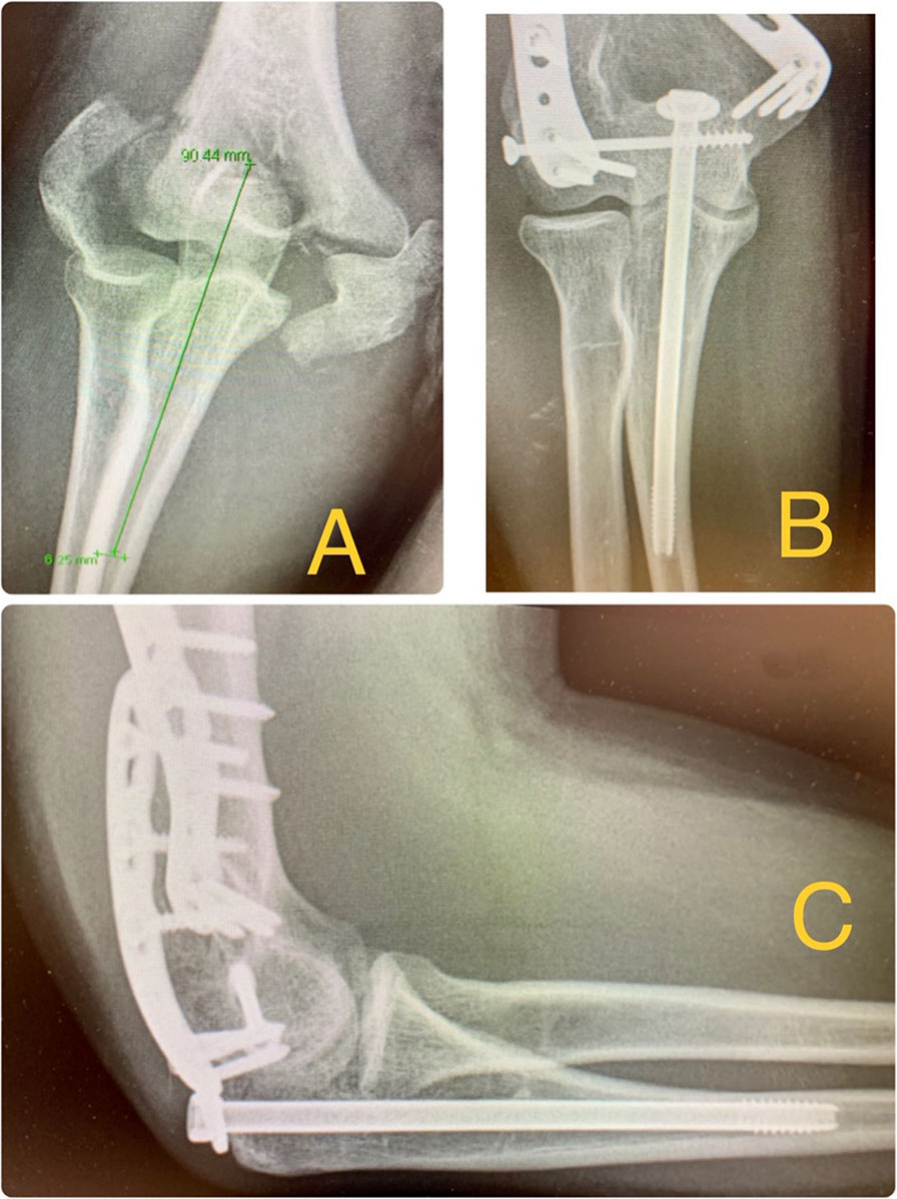
63 year old man who presented a conminuted supraintercondylear fracture of the humerus. Osteosynthesis of the fracture with two plates and a 6.5 mm thick and 110 mm long screw with washer for the olecranon osteotomy. **a** AP X-ray with the mesured ulna diaphysis diameter (6′25 mm) at 90 mm to the tip of the olecranon. **b** AP X-ray after 3 months where the consolidation of the osteotomy can be appreciated, the screw is long enough to achieve a good grip in the cortex of the ulnar diaphysis to get enough compression and stability. The medial deviation at the union of the medial and proximal 1/3 third (9–11 cm from the tip of the olecranon) can be observed in this X ray. This fact of the anatomy of the ulna contributes to the screw clamping. **c** Lateral X-ray after 3 months where the consolidation of the osteotomy can be appreciated, the screw is long enough to achieve a good grip in the cortex of the ulnar diaphysis to get enough compression and stability. The anterior deviation at the union of the medial and proximal 1/3 third (9–11 cm from the tip of the olecranon) can be observed in this X-ray. This fact of the anatomy of the ulna contributes to the screw clamping

**Fig. 3:**
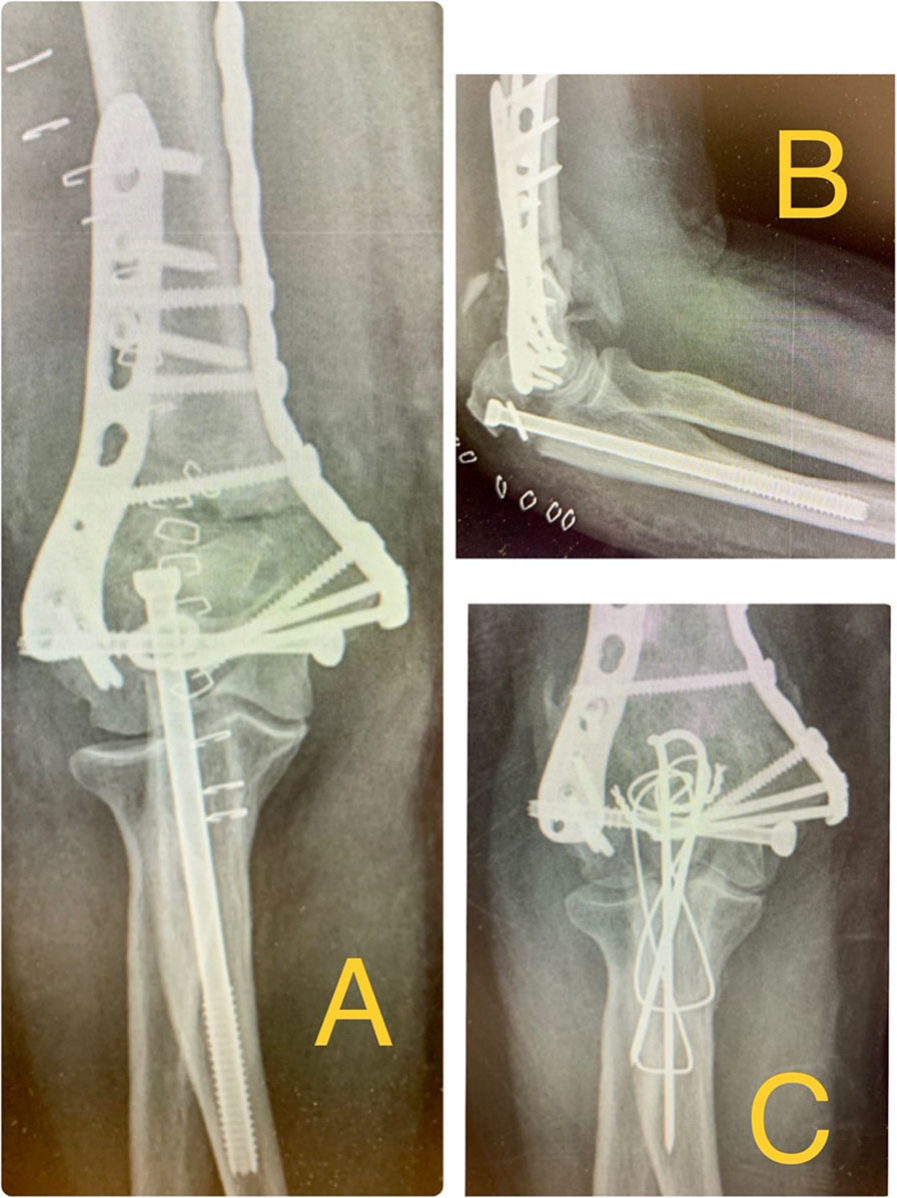
**a**, **b** 73 year old man who presented a conminuted supraintercondylear fracture of the humerus. Osteosynthesis of the fracture with two plates and a 6.5 mm thick and 120 mm long screw with washer for the olecranon osteotomy. X-ray on the first postoperative day, with acute failure of osteosynthesis with impaction of the screw into the medullary canal of the ulna. **c** Control X-ray at 2 months after revision surgery to a tension band cerclage, showing osteotomy consolidation and good functional result

**Fig. 4:**
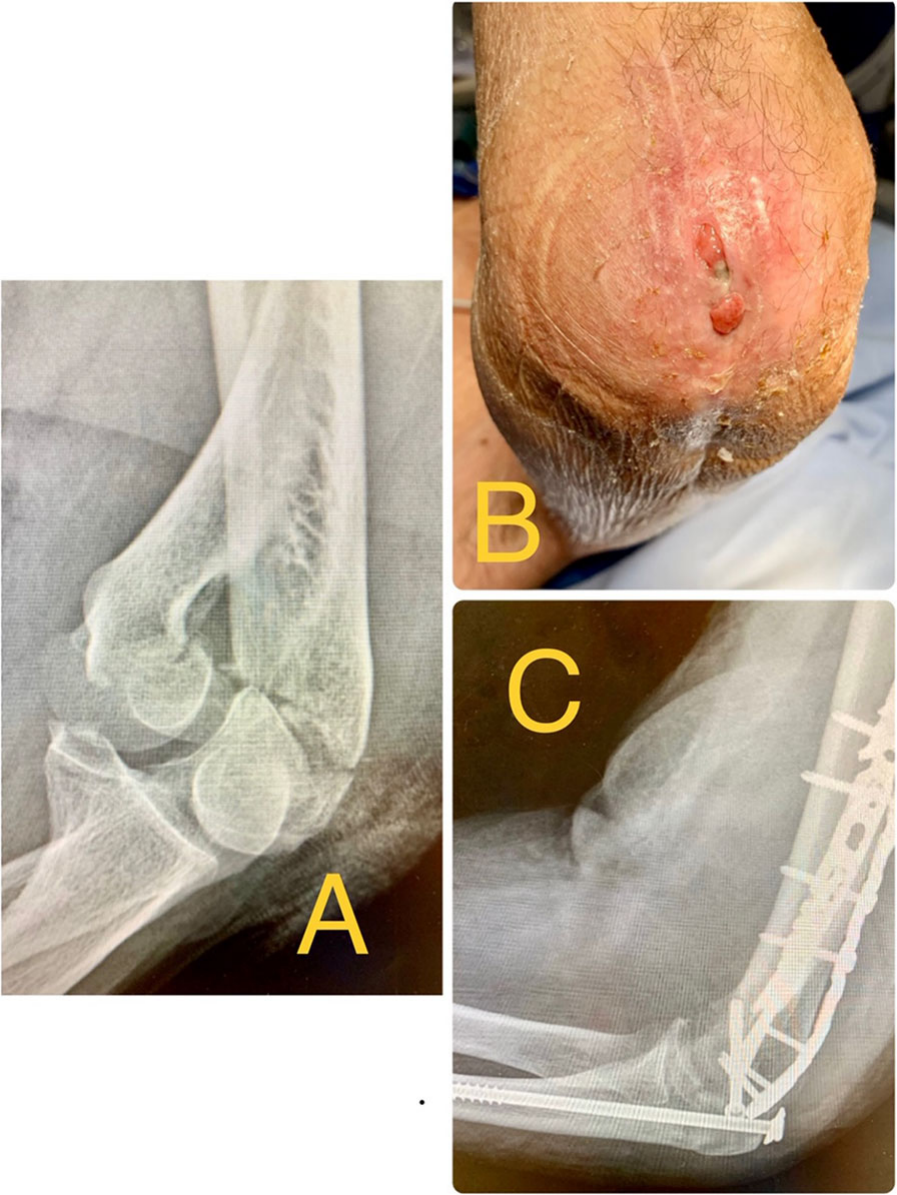
**a** 83 year old man who presented a conminuted supraintercondylear fracture of the humerus. **b** Wound dehiscence that required outpatient nursing cures every 48 h until enough bone callus was observed in most part of the ulna after 10 weeks; the wound healed well after the screw removal. **c** X-ray at 10 weeks after the first surgery. Osteosynthesis of the fracture with two plates and a 6.5 mm thick and 115 mm long screw with washer for the olecranon osteotomy. Enough bone callus was observed in most part of the ulna, considering the bone union stable to remove the screw

Some authors have used 4.5 or 6′5 mm diameter cortex screws, adding a tension band for reinforcement, with excellent results [5, 19]. We have only used one screw with a washer, even in older patients with more severe osteoporosis (Fig. 2, 3 and 4). It is essential that this 6.5 mm diameter cancellous and partially threaded long screw grips the ulnar diaphyseal cortex during several turns, thus achieving effective compression and good stability, even in older patients, without the need to add another reinforcement fixation system, and which allows early mobility. It has to be a long screw,102.3 mm on average, not finding differences between age and osteotomy consolidation.

In 2015 Woods (21) had already demonstrated that the fixing of an osteotomy with a 6.5 mm or 7.3 mm screw and washer was an effective and safe method.

The ulna medullary canal diaphysis diameter has been measured radiographically with a validated radiographic measurement system (Centricity Enterprise Web 3.0 DICOM conformance Memo (GE Medical Systems)), and we have also verified by measuring with this system the length and diameter of the screw in the x-ray and comparing it with the real size of the screw to check the validity of measurement (Fig. 1). Several authors have studied the anatomy of the ulna [1, 2, 12], especially to assess the use of endomedullary nails in osteosynthesis of diaphyseal ulnar fractures. The main length of the ulna in an adult is between 22 and 28 cm, it has a medial and anterior deviation approximately at the intersection of the medial and proximal 1/3 third, that is 9–11 cm from the tip of the olecranon. And the ulna medullary canal diaphysis diameter in the narrow area would be between 3 and 6 mm on a lateral radiograph and 3.5 and 7 mm on an anteroposterior radiograph.

Moreover, Bosman in 2020 [3] used 7′3 mm intramedullary screw fixation for simple, displaced fractures in 15 patients with excellent results. They used screws of enough length to engage the narrow marrow of the proximal ulnar diaphysis (typically 90–110) to get a stable fixation.

For these reasons, with a partially threaded 6.5 mm cancellous screw, we achieve fixation in the cortical diaphysis of the ulna of at least 0.5 or 1 cm, due to the narrow ulnar tunnel, thinner than the screw; Also because of the anteromedial curvature of the ulna, the straight screw will grip in the lateral and posterior cortex of the ulna (Fig. 2). But it is essential that the screw be long enough to provide stable fixation (102 mm of length on average in our study).

The different published studies of ulna medullary diaphysis diameter measurement and the use of screws for olecranon osteosynthesis, support our technique of osteosynthesis with a 6.5 mm screw and an average length of 102 mm [1–3, 12].

The use of cannulated screws has advantages; it makes it easier to check that we are centred in the ulnar diaphysis, makes the measurement of the screw easier, helps in the correct, fast, and straightforward insertion of the screw in both fragments at the end of the surgery. The V-shaped Chevron olecranon osteotomy increases the stability of the fixation over the horizontal osteotomy, as well as increasing bone contact surface between the fragments, favouring their consolidation [10, 17, 19, 21].

There are a series of cases published with up to 27% of re-interventions to remove the osteosynthesis materials with cerclages with Kirschner wires [13], as well as 10% non-unions using fixation with Kirschner wires or cortical screw and a reinforcement cerclage. Coles [5], in 2006, reported 100% consolidations of osteotomies fixed with a cortical screw and a dorsal cerclage, with two cases of early displacement that required early re-intervention and fixation with a plate and with 29% of the cases with olecranon osteosynthesis removal. Ring [17], in 2004, published excellent results with fixation with a tension band and fixation of the Kirschner wire to the anterior cortex of the ulna.

Our consolidation rate is 100% at 12 months, with two cases of delayed consolidation that consolidated at 6 and 12 months, respectively (Table 1). Previous clinical reports have published non-union or delayed-union in 10% of the cases [8, 9, 13].

In our study, 31% (8/26) of the patients requested the extraction of the osteosynthesis hardware, especially due to discomfort in the humeral plates. This percentage is comparable to that of other authors such as Ring [17] with 27% of the cases.

The younger patients are the ones that most request the hardware removal, mainly due to the discomfort in the plates inserted in the humerus, while this is rarely requested by elderly patients, being 28 years the average age of the patients in which the osteosynthesis hardware was removed.

There is a risk of skin necrosis and dehiscence of the wound in the proximal ulna, especially in the elderly, due to it is an area with practically subcutaneous bone [5, 7, 9, 13, 17, 21]. One of the advantages of using only one screw compared to plates, is that we can wait for the consolidation of the osteotomy with outpatient treatments, and after the radiographic consolidation, easily remove the screw, which makes the closure of the wound easier. This is normally not so easy with plates as there is much more metal that hinders wound healing (Fig. 4).

**Fig. 5:**
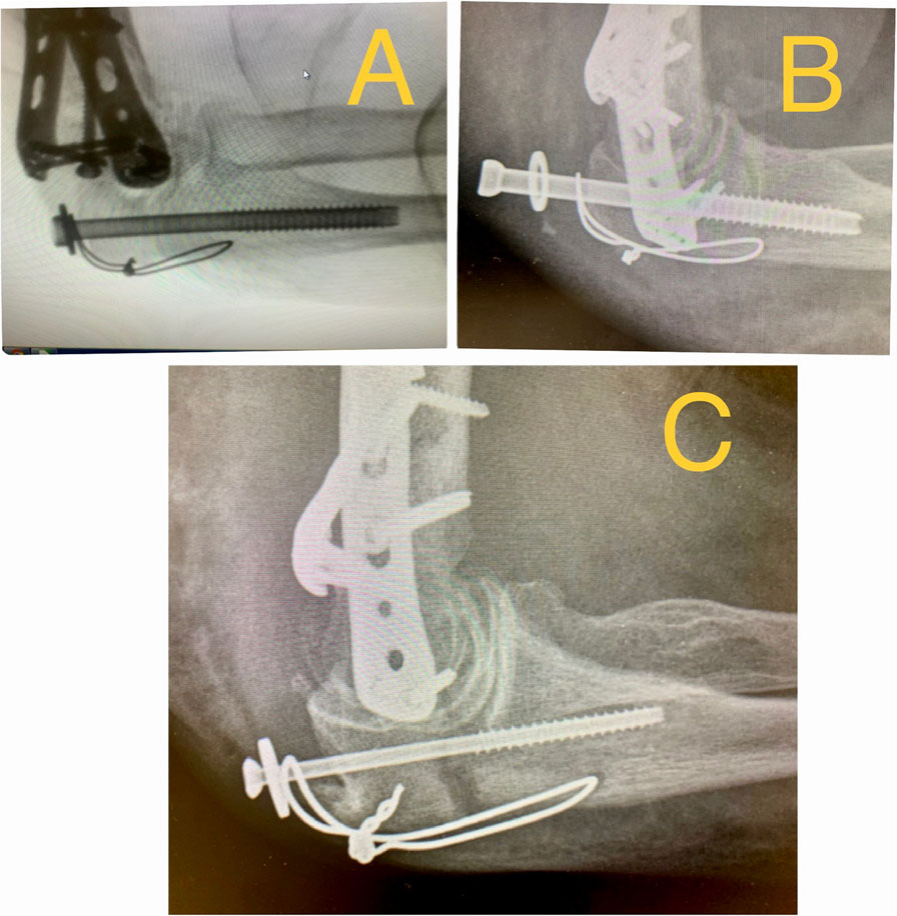
**a** Lateral intraoperative X-ray of 74 year old woman who presented a conminuted supraintercondylear fracture of the humerus, fixed with two plates and a 6′5 mm thick and 60 mm long screw with a washer and a cerclage for the olecranon osteotomy. The screw is not long enough to grip the cortical of the ulna canal diaphysis. **b** Same case; X-ray 5 weeks after the surgery with acute failure of the osteosynthesis of the olecranon osteotomy. In this case, we waited 4 more weeks until the osteotomy consolidation and then we removed the screw and cerclage. **c** Lateral X-ray of a 68 year old man with a supraintercondylear fracture treated with two plates in the humerus and a 4′5 mm thick and 42 mm long screw and a cerclage for the olecranon osteotomy. In this case an acute loss of reduction of the fixation of the osteotomy appeared, with a gap of more than 2 mm. The screw is not long neither thick enough to grip the cortical of the ulna canal diaphysis and provide enough stability

We began to use this technique in order to reduce the non-union and displacement rate of the wires with cerclages. We had also bad experiences with shorter or thinner screws despite adding a cerclage (Fig. 5). That is why we emphasize the importance of using 6′5 mm cancellous partial threaded screws and long enough to grip the ulnar diaphysis cortex; Woods [21] has already obtained good results with 6′5 or 7′3 mm screws of sufficient length to grip cortical bone of the ulnar shaft. We totally agree with Woods and in our experience, good distal cortical fixation is necessary to obtain good interfragmentary compression and enough stability.

We also looked for a fast and simple technique that would enable us to perform a stable fixation that would not require performing complex technical manoeuvres at the end of a long and demanding surgery. This technique allows us to meet these objectives with excellent results. Apart from the study by Woods in 2015 [21], we have not found any other clinical publication that fixes the osteotomy with only one screw and a washer.

We are aware that the study has several weaknesses, as it is a retrospective study with no control group to compare another technique. No definitive conclusions can be drawn as the number of patients is limited. This study is basically radiographic and not clinical.

But it also has its strengths: all the patients have been operated on by the same surgeon, expert in shoulder and elbow surgery, the patients have been followed-up for at least one year, and always until the consolidation of the osteotomy, and the diameter of the ulnar canal has been measured in all patients.

Although 26 patients is not a large number, it is sufficient to validate the technique in these, quite uncommon, fractures.

## Conclusion

Chevron shape olecranon osteotomy gets the best exposure of the articular surface in a supraintercondylar humeral fracture. Regardless the type of osteotomy fixation, a stable and secure fixation has to be achieved to allow early elbow motion and minimize risks and complications. Ostetomy fixation with a 6′5 mm cancelous partial threaded screw and washer was effective, allowing an excellent evolution of the patients with a very high consolidation percentage and with complication rates similar to or lower than other fixation methods published. Long enough screws must be used to get a good cortical grip with enough stability.
